# The Use of Opium in Acute Internal Inflammations

**Published:** 1842-03

**Authors:** Robert Christison

**Affiliations:** Professor of Materia Medica in the University of Edinburgh


*The use of Opium in Acute Internal Inflammations.* By Robert Christison, M. D., Professor of Materia Medica in the University of Edinburgh.—1. The varieties of internal inflammation which best exemplify this action of opium, when given singly, are the inflammations of the mucous membranes. Of such diseases there are at least four which may often be thus successfully treated without almost any other remedy, namely, coryza, catarrh, influenza, and dysentery.

As to *Coryza,*—most persons on feeling its approach, are content to submit without a struggle to the infliction, and eschewing alike physic and the physician, let their "cold in the head" take its own way. But few would do so, were they aware how easily and how agreeably so tormenting a visitor may for the most part be got rid of. For twenty years I have been accustomed to see it stopped at once by a full opiate given on the first day of its appearance. Let the patient avoid food after dinner, use liquids sparingly, take a full dose of muriate of morphia, or Battley's solution, at bedtime, and breakfast before getting up in the morning,—and he will then commonly find the secretion of the nostrils permanently inspissated, and the complaint either gone entirely, or at any rate no longer a source of particular annoyance.

During the same period I have often seen a common *catarrh without fever* cut short in like manner, if taken on the first, second, or perhaps even the third day. Or more generally it seems to pass at once over the intervening stages to that in which thick sputa are coughed or hawked up without labor, and without irritation in the chest or windpipe. *Febrile catarrh* too may be checked abruptly in the same way, if patient and physician are lucky enough to meet during the first or the second day at farthest. But here probably the next mode of employing opium is fully more successful. I have also repeatedly seen epidemic *influenza* thus checked at the outset,—the local inflammation vanishing, while the strange lassitude, listlessness, and ennui, so characteristic of this disorder, went on as usual for some days during convalescence.

Although an authority so recent and so eminent as Mr. Pereira states, that "in *dysentery,* opium can only be used beneficially in the latter stages, and then with great caution" (*Mat. Med.* 1303), this is another and more formidable malady, which, if I mistake not, may be added to the mucous inflammations capable of being cut short in the early stage by opium. I doubt whether Mr. Pereira's doctrine will be received at all as a general proposition. There is not a better way of treating an ordinary mild dysentery at the commencement, than by the familiar practice of alternate opiates and laxatives. But—what is more connected with the matter now chiefly under review—in severe epidemic dysentery, as it occurs at times in this country, the cure when early begun, may be commonly trusted, if I may rely on my own experience, to opium alone,—not used, however, "with great caution," but given boldly and often.

2. The method of using opium along with ipecacuan as a sedative anodyne, and sudorific, in the early stage of acute inflammations, is probably applicable to a considerable number of diseases of the kind. Those alone in which I have employed it are common sore throat, catarrh, and acute rheumatism.

From frequent observation, I am inclined to think that there are few cases of *acute cynanche tonsillaris,* which may not be cut short at the outset by this treatment, if they are subjected to it about the close of the first, or at all events before the close of the second day. Of several instances I have met with to this effect, the following are examples, A lady, rather subject to febrile cynanche tonsillaris, was attacked with rigors in the evening, and sore throat during the night. Next day the right tonsil was much enlarged and red, and the posterior palate and velum red and thickened,—the pulse at the same time being one hundred and twenty, and the febrile oppression considerable. In the evening, and therefore within the first twenty-four hours, ten grains of Dover's powder were given at intervals of half an hour, till half a drachm was taken. Perspiration which soon broke out gently, was kept up for fifteen hours by warm drink. In twenty hours after the powders were taken, the pulse was seventy, the pain in swallowing gone, and the swelling and redness insignificant; and on the subsequent morning she was able to leave her bed. This attack happened eight years ago at least, and the disease has not returned since. A lady about thirty-eight, long a martyr to cynanche tonsillaris,—which, in particular for three years before, had returned during the winter, ended in abscess of one or both tonsils, occasioned great and protracted torture, and had left chronic enlargement of these glands,—was attacked in the same way for the fourth time. When I first saw her on the fourth day of the disease, the tonsils, greatly enlarged, especially on the left side, blocked up nearly the whole throat. She spoke with much difficulty, could not swallow without long, painful, and repeated efforts, and breathed noisily and with labor. The pulse was one hundred and sixteen, full and soft. The febrile oppression and anxiety were great. A large blister had been applied to the throat, and purgatives taken freely, but without the slightest benefit. The attack seemed too far advanced to be arrested. But as fluctuation could not be detected in the larger tonsil, Dover's powder was ordered as in the former case. Sweating ensued; ere long, warm drink could be swallowed with no great difficulty: and when the perspiration had thus been kept up for twenty-four hours, the pulse had fallen to sixty-four, the swelling of both tonsils had materially subsided, and the febrile anxiety had ceased, together with the local uneasiness in a great measure. In two days more there was little complaint left except weakness. In the course of time, the chronic enlargement of the tonsils disappeared; and this lady, like the last, has never had another attack, though five years have elapsed since the one now described. The tendency to cynanche tonsillaris, certainly seems to grow with its repetition in a severe form; and I have met with other instances besides these two, where an abrupt early cure seemed to break both the attack and the liability. The only objection, or rather obstacle, to this plan of cure is, that patients unluckily will seldom put themselves in the way of profiting by it; as the practitioner does not often see febrile sore throat, till the chief question he has to consider under the head of treatment is, whether an abscess in the tonsil is to be opened by nature or the lancet.

I have seen *febrile catarrh* quickly arrested in the same way in persons prone to it. But the circumstances have been so similar to those just described, that a detail of them here seems unnecessary.

Whether *acute rheumatism* may also be treated successfully by the like means, is a matter of doubt. At the period at which medical men generally first come in contact with a case of it, sweating by Dover's powder will not succeed singly. But this is a powerful and speedy measure, if immediately preceded by general blood-letting. Perhaps, then, the present topic would be better deferred, and taken up under the next head. But since it is the action of opium as a sudorific, which seems here the source of its curative influence, while in the third mode of using it for arresting acute inflammatory diseases of this kind of action is not at all essential, the treatment of rheumatism will be more conveniently disposed of in the present place.

There are physicians of undoubted authority, who maintain the acute rheumatism can scarcely be cut short,—that, do what one may, the patient must endure towards three weeks of agony, and three weeks more of stiffness, weakness, and occasional aches. I am convinced, however, that, at least in young adults of sound constitution, a genuine acute attack may often be put an end to in a few days, by first drawing blood very freely to the approach of faintness, and then giving Dover's powder instantly afterwards in the way mentioned above for inflammatory sore throat. I have often observed, that after sweating has been thus brought out and kept steadily up for thirty-six or forty-eight hours, the pulse fell to the natural standard from nearly double that rate, the white thickly-furred tongue began to clean, the pains and redness gave place to numbness and want of power, recovery went on afterwards swiftly and without interruption, and the patient was able to leave his bed in a week. The chief conditions for success are, that the bowels, previously opened if necessary, shall be let alone till the sweating is well over; that blood be drawn both largely and to approaching syncope; that the powders be given immediately afterwards, before the circulation recovers its state of excitement, which it will otherwise soon do; that the treatment shall be enforced before the close of the fourth day, but earlier if possible; and that the case shall be real acute rheumatism, not one of the sub-acute form, or gouty rheumatism, as it is called. Where for some days previously the local inflammation has been shifting from joint to joint, with irregularly intermitting fever, I have once or twice tried to do without the preliminary blood-letting, but have not succeeded. In one instance, that of a stout young cabinet-maker, copious sweating begun on the third day without blood-letting, and maintained for eight-and-forty hours, was of no avail; but when repeated after one free evacuation of blood, it put an end to the disease abruptly in the way just described.

3. The treatment of acute internal inflammations by opium after blood-letting is now currently used by some practitioners. But since, notwithstanding its efficacy, it is still not generally practised, it seems to need the support of farther testimony than it has received from the few who have taken public notice of it.

This mode of cure consists in withdrawing blood very freely from the arm till faintness approaches, just as in the ordinary way of treating acute inflammations,—and then giving a large opiate, with a view to bring on sleep or the calm reverie which in some people takes the place of sleep. The result is, that, on the patient awaking, the general fever and local inflammation are found to be subdued and broken,—generally at once, but sometimes not till the repetition of the practice in twelve or twenty-four hours.

The conditions for successfully employing opium after blood-letting are nearly the same with those for using Dover's powder in rheumatism. It is essential that the disease to be subdued be in its early stage; that a deep impression be made on it by blood being withdrawn both freely, and to the approach of faintness; and that the opium be given largely and immediately so as to anticipate the renewal of reaction. Sweating sometimes ensues, but it is not at all a necessary condition for success. The particular preparation of opium to be used, is perhaps of no great consequence. I prefer the solution of muriate of morphia, or, failing that, the sedative solution of Battley. The dose of the former should not be less than forty minims, for an adult male, and for others in proportion; and the dose of Battley's solution, which is certainly not so strong as its maker represents, should not be less than twenty-five or thirty minims. Some conceive this treatment applicable only to inflammation of membranous surfaces, not to that of parenchymatous textures. I do not know any positive facts either on one side or the other of this question; but the statement is doubtful, if it be meant to apply to acute parenchymatous inflammations in their early stage, If pneumonia be regarded as inflammation of a parenchymatous tissue, which, although a common mode of viewing it, is rather incorrect,—then, there can be no doubt that in this particular instance the treatment is most effectual on many occasions.—*Braithwaite's Retrospect Pract. Med. and Surg.* No. 3, from *Edin. Monthly Jour. of Med. Sci.* Feb. 1841.

